# Evaluating the effectiveness of satellite image super-resolution for road quality monitoring

**DOI:** 10.1038/s41598-026-47749-3

**Published:** 2026-04-16

**Authors:** Aaron Thegeya, Thomas Mitterling, Clifford Njoroge, Arturo Martinez, Yohan Iddawela, Joseph Albert Niño Bulan, Ron Lester Durante, Oshean Lee Garonita, Jayzon Mag-atas

**Affiliations:** 1 World Data Lab, Vienna, Austria; 2 Asian Development Bank, Manila, Philippines

**Keywords:** Super-resolution, Road quality, Road maintenance, Sustainable development goals, Remote sensing, Deep learning, Engineering, Mathematics and computing

## Abstract

Monitoring road infrastructure is essential for accessibility and economic development, yet data scarcity remains a major constraint in many developing countries. Satellite-based deep learning approaches provide a scalable alternative, but the coarse resolution of freely available imagery may limit performance. This study evaluates whether generative adversarial network-based super-resolution can improve road roughness classification from Sentinel-2 imagery. Using a national dataset of 124,462 road segments from the Philippines, we compare classification results from native medium-resolution inputs with high-resolution images generated using real-ESRGAN. Despite clear visual improvements and higher perceptual quality scores (PSNR increasing from 15.3 to 16.7 dB), super-resolution did not improve predictive performance. Binary classification accuracy differed by less than 0.5% points, while four-class accuracy declined by 2 to 4% points when using super-resolved imagery. Most errors occurred between adjacent roughness categories, and super-resolution did not reduce this confusion. In contrast, combining native imagery with environmental covariates, including temperature, precipitation, slope, and population density, substantially improved results, achieving 85% binary and 71% four-class accuracy. These findings indicate that preserving original spectral information and incorporating contextual variables is more effective than generative image enhancement for satellite-based road quality assessment in data-sparse settings.

**Supplementary Information:**

The online version contains supplementary material available at 10.1038/s41598-026-47749-3.

## Introduction

The longevity of infrastructure is important for realizing its intended benefits, making asset maintenance essential. In general, neglecting infrastructure leads to asset degradation, which negatively impacts the economy and results in higher reconstruction costs over time^1^. Road infrastructure degradation can disrupt economic activities, impede the flow of goods and services, and hinder labor mobility. However, maintaining a road network requires substantial resources, and costs are significantly higher for roads that have fallen into severe disrepair versus requiring only minor maintenance. Therefore, identifying roads in need of maintenance and deploying appropriate resources is key to minimizing these costs. In low- and middle-income countries, limited public resources often make road quality monitoring and maintenance particularly challenging.

In the case of the Philippines, the design life for concrete roads is set at 20 years, while 10 years is set for asphalt roads, as specified by the Department of Public Works and Highways^2^. However, to maintain serviceability and avoid costly reconstruction or replacement, roads should ideally not be allowed to deteriorate to the end of their design life. The Department of Public Works and Highways (DPWH) is primarily responsible for major maintenance of national roads, typically every 10 years for concrete pavements and every 5 years for asphalt^3^. Local government units oversee the maintenance of local roads. Nevertheless, the actual need for maintenance can vary significantly based on external conditions, such as natural environmental factors.

In other countries, there have been research focusing on ground-level road condition analysis and automated maintenance. Various studies explore vision-based crack detection on edge devices and mobile robots^4,5^, as well as autonomous crack repair systems that seal or trace cracks using robotics^6–9^. These efforts – along with recent surveys of road AI and robotic technologies^10,11^– illustrate the growing interest in automating road monitoring and maintenance.

While regular maintenance and monitoring are crucial for maximizing the socioeconomic benefits of transport infrastructure, these are often underfunded^12^. Many countries spend only 20–50% of the required maintenance budget, and the high cost of collecting road condition data often limits its availability, especially in resource-constrained settings. In the Philippines, collecting road condition data usually involves the use of equipment such as sensors, vehicles’ onboard devices, and audio and video streams^13^.

The absence of reliable data on road conditions^14^ poses a significant challenge to timely and effective infrastructure interventions. Without such data, it becomes difficult to identify which roads require rehabilitation, potentially delaying maintenance and worsening accessibility. This issue is especially pronounced in rural areas, where data collection efforts often prioritize urban regions. As a result, rural road conditions are frequently underreported or overlooked. Recognizing this gap, Sustainable Development Goal (SDG) 9.1.1, the Rural Accessibility Index (RAI), specifically targets rural development. It measures the proportion of the rural population living within 2 km of an all-season road, which in some contexts is determined by road roughness^14^. This indicator underscores the importance of inclusive infrastructure planning that ensures equitable access for all, particularly in underserved rural communities. However, RAI data remain sparse. In Asia and the Pacific, less than 7% of economies have data on RAI based on the United Nations Statistics Division’s Global Sustainable Development Goal Database.

Recent advances in satellite data and machine learning (ML) show the potential for reducing costs and increasing the frequency of road quality monitoring, especially in rural areas. Satellite imagery offers regular coverage of most global locations and can be used to map road conditions by training machine learning algorithms to predict road quality. Thus, leveraging satellite data and ML provides a cost-effective method for monitoring road quality, enabling better planning and more efficient allocation of limited maintenance resources.

The growing interest in road quality identification through ML has led to significant advancements, particularly using Convolutional Neural Networks (CNNs). Multiple studies have successfully leveraged Convolutional Neural Networks for classifying road conditions based on satellite imagery, with many also addressing challenges related to data resolution and quality. Cadamuro, Muhebwa, and Taneja^15^ developed a Convolutional Neural Networks to predict road quality in Kenya, using International Roughness Index (IRI) data as a proxy for road quality. Their study analyzed 1,150 km of road data combined with 50-cm resolution satellite imagery, employing Convolutional Neural Network variants such as AlexNet and Visual Geometry Group (VGG) to achieve 87% accuracy for binary classification. Oshri et al.^16^ examined infrastructure quality across Africa using Sentinel-1 and Landsat-8 imagery combined with survey data, achieving 70.5% accuracy with a ResNet model. Similarly, Abdelaziz et al.^17^ demonstrated that standard Convolutional Neural Networks outperformed linear regression for pavement performance prediction, with ResNet architectures proving particularly efficient for classifying distress in highway images.

However, a critical challenge remains: the trade-off between image resolution and acquisition cost. High-resolution imagery yields better predictions but is cost-prohibitive for many developing nations. To address this, the remote sensing community has largely converged on using Generative Adversarial Networks (GANs) for satellite image super-resolution to enhance the resolution of freely available images, thereby bridging the gap between freely available medium-resolution imagery and costly commercial data. One of the most common approaches is the Super-Resolution GAN (SRGAN) framework.

SRGAN-style architectures have inspired a significant wave of research in remote sensing super-resolution because they can recover fine spatial details crucial for analyzing land cover and infrastructure^18^. Unlike traditional interpolation, GANs employ adversarial training, pitting a generator against a discriminator, to synthesize realistic high-frequency details. This results in imagery that is spatially coherent and perceptually plausible. While newer architectures like diffusion models are emerging, GAN-based approaches remain a popular choice for operational production settings and are actively explored for their ability to balance reconstruction fidelity with perceptual realism. Consequently, GANs represent the established baseline for assessing whether synthetic resolution enhancement can benefit downstream tasks.

Despite their status as the standard for visual enhancement, the utility of GAN-based super-resolution for general road roughness classification remains unclear. While some studies suggest improvements in detecting small objects, others indicate that super-resolution improves aesthetics without consistently boosting the discriminative power of classification networks^19^. For instance, Jaffe et al.^19^ found that while SR minimized perceptual loss, it did not necessarily aid classification, a finding echoed by other studies suggesting that accuracy gains from resolution plateau beyond a certain point^20,21^.

This divergence highlights a critical research gap: does visual fidelity from super-resolution translate into better machine interpretability for road roughness? Existing studies often separate super-resolution from classification or test it on highly localized datasets (e.g., drone footage of cracks) rather than large-scale satellite monitoring.

To address this gap, this study investigates whether integrating GAN-based super-resolution with deep learning models enhances road quality classification accuracy across diverse environments. Building on the work of Thegeya et al.^22^, we scale the analysis from previous subsampled approaches to a national-scale dataset of road segments in the Philippines. We employ a robust PyTorch-based framework to handle the full dataset (over 124,000 observations), overcoming the memory and processing limitations of previous high-level API implementations. By systematically comparing the performance of models trained on original medium-resolution Sentinel-2 imagery against those trained on super-resolved outputs, this paper tests the hypothesis that generative enhancement improves predictive utility for infrastructure monitoring in resource-constrained settings.

In doing so, we created an image classification pipeline involving three key steps. First, we performed dataset preparation by constructing a robust dataset using remotely sensed data of road segments obtained from Google Earth. Second, we conducted co-registration, where road segment data were matched to IRI ratings obtained from survey data and formatted into objects for training. Third, we trained a classification model.

In addition to classifying image data, we implemented a combined model architecture to integrate environmental context. This architecture consists of three primary components.

1. **Convolutional neural network (CNN)**: This component processes road image segments by employing convolutional layers to extract visual features.
2. **Sequential linear neural network (SLNN)**: This component processes tabular data related to the immediate geographical neighborhood of a given road segment, including data on temperature, precipitation, gradient, and population density.
3. **Combined sequential layer**: The outputs from both components are concatenated and fed into a final fully connected layer equipped with Linear Batch Normalization for regularization and Dropout to prevent overfitting, effectively classifying the combined inputs into an IRI rating.

By comparing the performance of models trained on original medium-resolution Sentinel-2 imagery against those trained on super-resolved outputs, this paper tests the hypothesis that generative enhancement improves predictive utility for infrastructure monitoring in resource-constrained settings.

## Methods

**Fig. 1:**
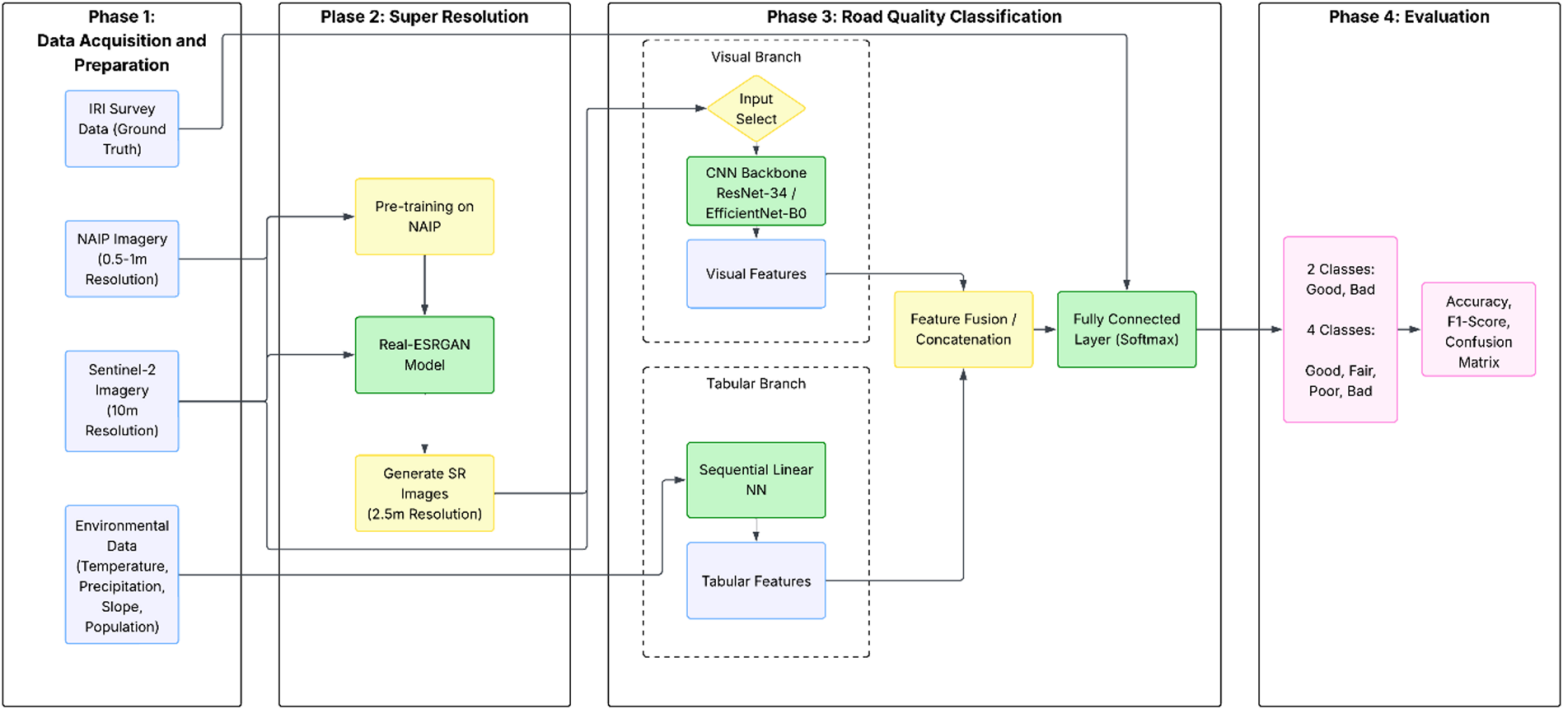
Methods framework.

To evaluate the impact of super-resolution on road quality assessment, we employed a multi-stage methodological framework (Fig. 1). The workflow was implemented sequentially rather than end-to-end. Specifically, the Real-ESRGAN super-resolution model was first fine-tuned on paired Sentinel-2 and NAIP imagery, and the resulting super-resolved images were then used as inputs for separately trained downstream deep learning road quality classification models. This design was chosen to isolate the effect of super-resolution as a preprocessing step on classification performance. Comprehensive model specifications, hyperparameters, and computational environment details are provided in Supplementary Information S1.

### Data acquisition and preparation

The study utilized the international roughness index (IRI) as the ground truth proxy for road quality. The IRI measures the cumulative suspension motion of a vehicle divided by the distance travelled and is typically standardized at 80 km/h. The dataset sourced from the World Bank covers national roads across 15 regions in the Philippines, including Luzon, Visayas, and Mindanao, as of 2019. This dataset comprises 124,462 geolocated road segments of approximately 100 m in length. We categorized the IRI values into four standard quality classes: Good, Fair, Poor, and Bad. The dataset exhibits class imbalance, with Bad roads comprising 31.5% of observations and Fair roads comprising 12.7%, which necessitated the use of class-weighted loss functions during training.

We acquired two types of satellite imagery for the super-resolution experiment. Sentinel-2 Harmonized Multispectral Instrument data served as the medium-resolution baseline input at 10 m per pixel. We generated a cloud-free mosaic for each road segment using a 90-day temporal window centered on the IRI survey date. For the high-resolution training targets, we used imagery from the United States National Agriculture Imagery Program (NAIP) at a ground sampling distance of 0.5 to 1 m per pixel. The NAIP data provided the high-frequency spatial details, such as lane markings and pavement texture, required to supervise the GAN training process. Additionally, we incorporated environmental variables known to influence road degradation, including mean annual temperature, precipitation, slope gradient, and population density, which were spatially joined to the road segment coordinates.

### Super-resolution strategy

To super-resolve Sentinel-2 images, we employed a Generative Adversarial Network (GAN). GAN-based approaches have emerged as the operational standard for remote sensing super-resolution because they balance reconstruction fidelity with perceptual realism. Unlike deterministic approaches (e.g., minimizing Mean Squared Error), which often produce overly smooth textures, GANs utilize adversarial training to synthesize plausible high-frequency details required for infrastructure analysis.

We utilized the Real-world Enhanced Super-Resolution General Adversarial Network (Real-ESRGAN) architecture^23^, a robust variation of the SRGAN. The Real-ESRGAN was selected specifically for its ability to handle “blind” super-resolution, where the degradation kernel (blur, noise, and downsampling method) of the input image is unknown or complex, as is typical with atmospherically corrected satellite imagery.

**Fig. 2:**
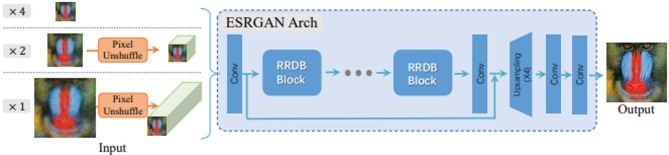
Real enhanced super-resolution generative adversarial networks architecture. From: Xintao Wang. 2021. “Real-ESRGAN: Training Real-World Blind Super-Resolution with Pure Synthetic Data”.

The network consists of a generator ($\:G$) and a discriminator ($\:D$) trained in a zero-sum game (see Fig. 2). The discriminator maximizes the probability of correctly classifying real images ($\:x$) versus synthetic images ($\:G$($\:z$)), while the generator minimizes the probability of detection. Unlike standard SRGANs that use a Visual Geometry Group-style classifier, Real-ESRGAN employs a U-Net Discriminator with Spectral Normalization. This allows the model to assess realism at both the global scale (structural consistency) and the local pixel scale (texture details), stabilizing the training process. Meanwhile, the Generator is built upon Residual-in-Residual Dense Blocks (RRDB); this network enables deep feature extraction without the vanishing gradient problem. It upsamples the 10 m Sentinel-2 input to a target resolution of 2.5 m (a scaling factor of 4).

The training process is formulated as a zero-sum minimax game. The discriminator attempts to maximize the probability of correctly classifying real high-resolution images ($\:x$) versus synthetic super-resolved images ($\:G$($\:z$)). The objective function for the discriminator is:

1 $$\:maximize{\mathbb{\:}\mathbb{E}}_{x}\left[\mathrm{log}D\:\left(x\right)\right]+\:{\mathbb{E}}_{z}\left[\mathrm{log}\left(1-D\left(G\left(z\right)\right)\right)\right]$$

Conversely, the generator aims to deceive the discriminator by minimizing the probability that its output is detected as fake:

2 $$\:minimize{\mathbb{\:}\mathbb{E}}_{z}\left[\mathrm{log}\left(1-D\left(G\left(z\right)\right)\right)\right]$$

To prevent the introduction of artifacts, the adversarial loss is typically combined with a perceptual loss (based on Visual Geometry Group-19 feature maps) and an L1 pixel-wise loss during optimization.

We adopted a transfer learning strategy to address the lack of high-resolution ground truth in the Philippines. We initialized the model with weights pre-trained on generic image datasets^20^ and fine-tuned it using 133,373 paired observations of road sections from the United States. These pairs consisted of high-resolution NAIP imagery (ground truth) and corresponding medium-resolution Sentinel-2 imagery. Fine-tuning was performed for 100,000 iterations using the Adam optimizer. To simulate the complex degradations found in satellite data, the training pipeline included a high-order degradation model involving random sinc filters, Gaussian blur, and noise injection. Further information on model specifications, hyperparameters, and computational environment details are provided in Supplementary Information S1.

The super-resolution stage was not jointly optimized with the downstream classification networks. After fine-tuning, the Real-ESRGAN model was applied to the Philippine Sentinel-2 dataset to generate a fixed set of super-resolved images. The classification models were then trained separately on either the original Sentinel-2 imagery or these generated images. We adopted this sequential strategy because the aim of the study was to test whether a widely used GAN-based super-resolution method improves downstream road roughness classification, rather than to develop a task-specific end-to-end architecture.

**Fig. 3:**
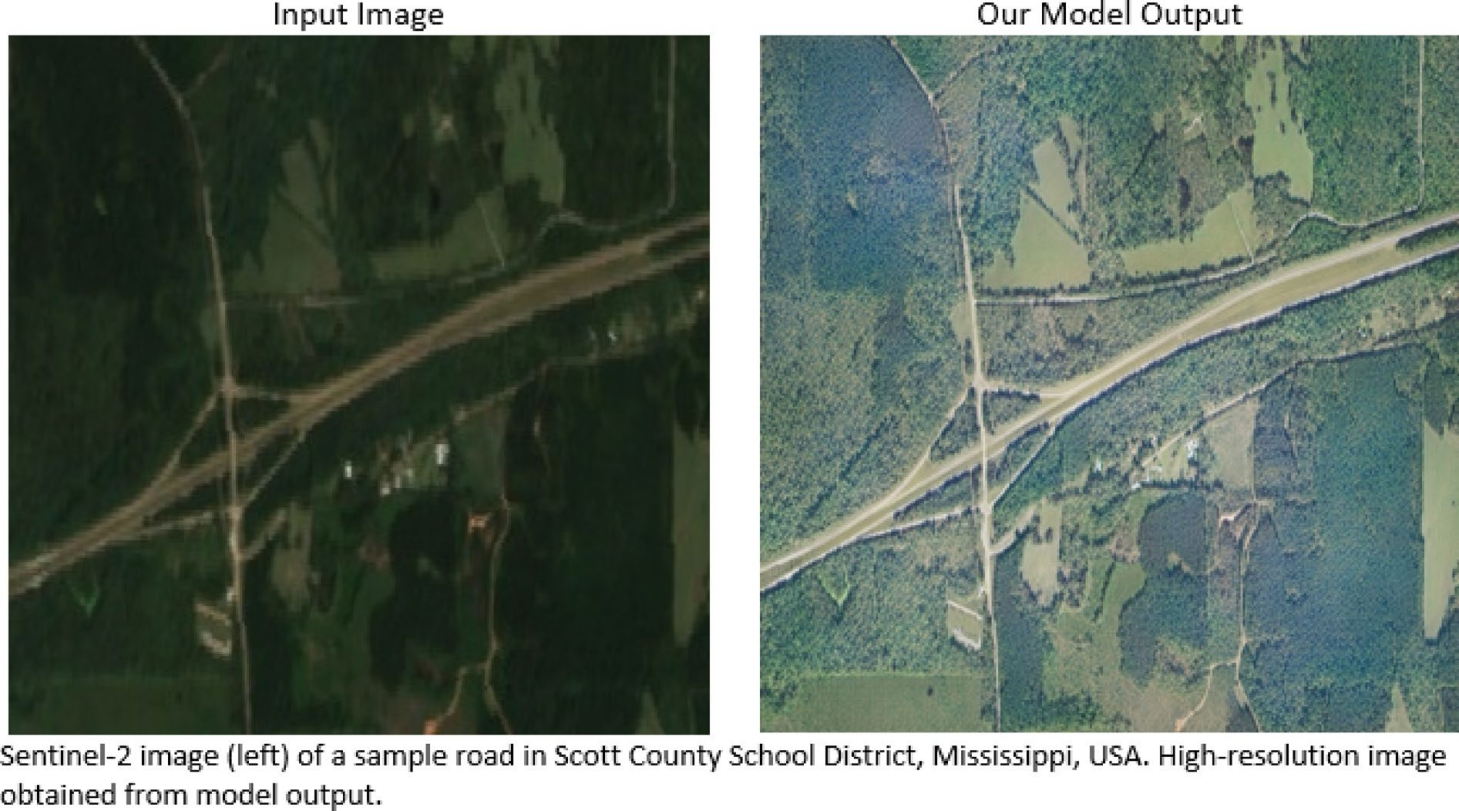
Comparison of low-resolution Sentinel-2 image and generated enhanced super-resolution general adversarial network image. Sentinel-2 image (left) of a sample road in Scott County School District, Mississippi, USA. High-resolution image obtained from model output. Source: Input image downloaded from Google Earth Engine: https://developers.google.com/earth-engine/datasets/catalog/COPERNICUS_S2_SR.

**Fig. 4:**
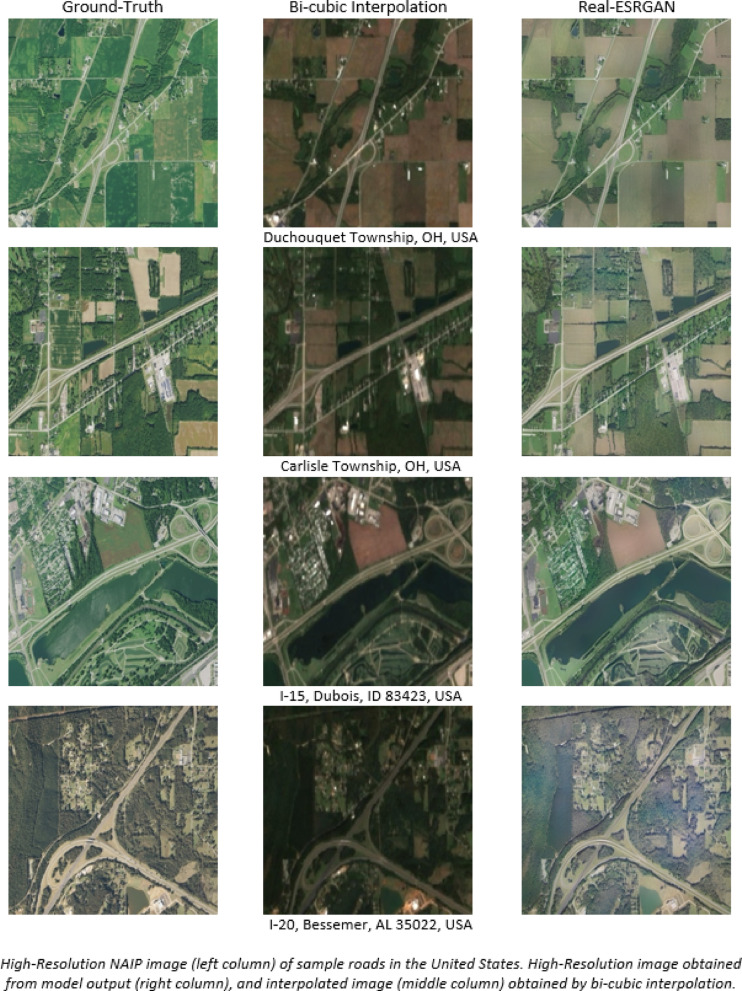
Comparison of high-resolution images. High-Resolution NAIP image (left column) of sample roads in the United States. High-Resolution image obtained from model output (right column), and interpolated image (middle column) obtained by bi-cubic interpolation. Source: NAIP images downloaded from Google Earth Engine: https://developers.google.com/earth-engine/datasets/catalog/USDA_NAIP_DOQQ.

To validate the model, we compared the Real-ESRGAN output against a baseline generated via bi-cubic interpolation. Figure 3 illustrates the visual enhancement of a sample road in the US, while Fig. 4 shows a comparison of three high-resolution images: a ground-truth image of NAIP data (at a scale of 1 m per pixel), an image generated by the Real-ESRGAN, and an image generated using bi-cubic interpolation.

To assess fidelity, Fig. 4 presents a side-by-side comparison of three high-resolution images: the ground-truth NAIP image (1 m/pixel), the image generated by the Real-ESRGAN, and the image generated using bi-cubic interpolation. Visually, the Real-ESRGAN image is significantly sharper than the bi-cubic interpolation, recovering distinct lane geometries that are otherwise blurred.

We subsequently evaluated performance using the peak signal-to-noise ratio (PSNR). The optimization target for super-resolution is the minimization of the Mean Squared Error (MSE) between the super-resolved image and the ground truth, which is equivalent to maximizing PSNR:

3 $$\:PSNR=10{log}_{10}\left(\frac{{R}^{2}}{MSE}\right)$$

where $\:R$ is the maximum pixel fluctuation (e.g., 255 for 8-bit data). Analysis of 100 random validation images yielded an average PSNR of 17.12 dB for Real-ESRGAN, compared to 15.18 dB for bi-cubic interpolation. While PSNR confirms superior reconstruction fidelity, we acknowledge its limitations; it is sensitive to sub-pixel misalignment and favors smooth images over texturally rich ones.

**Fig. 5:**
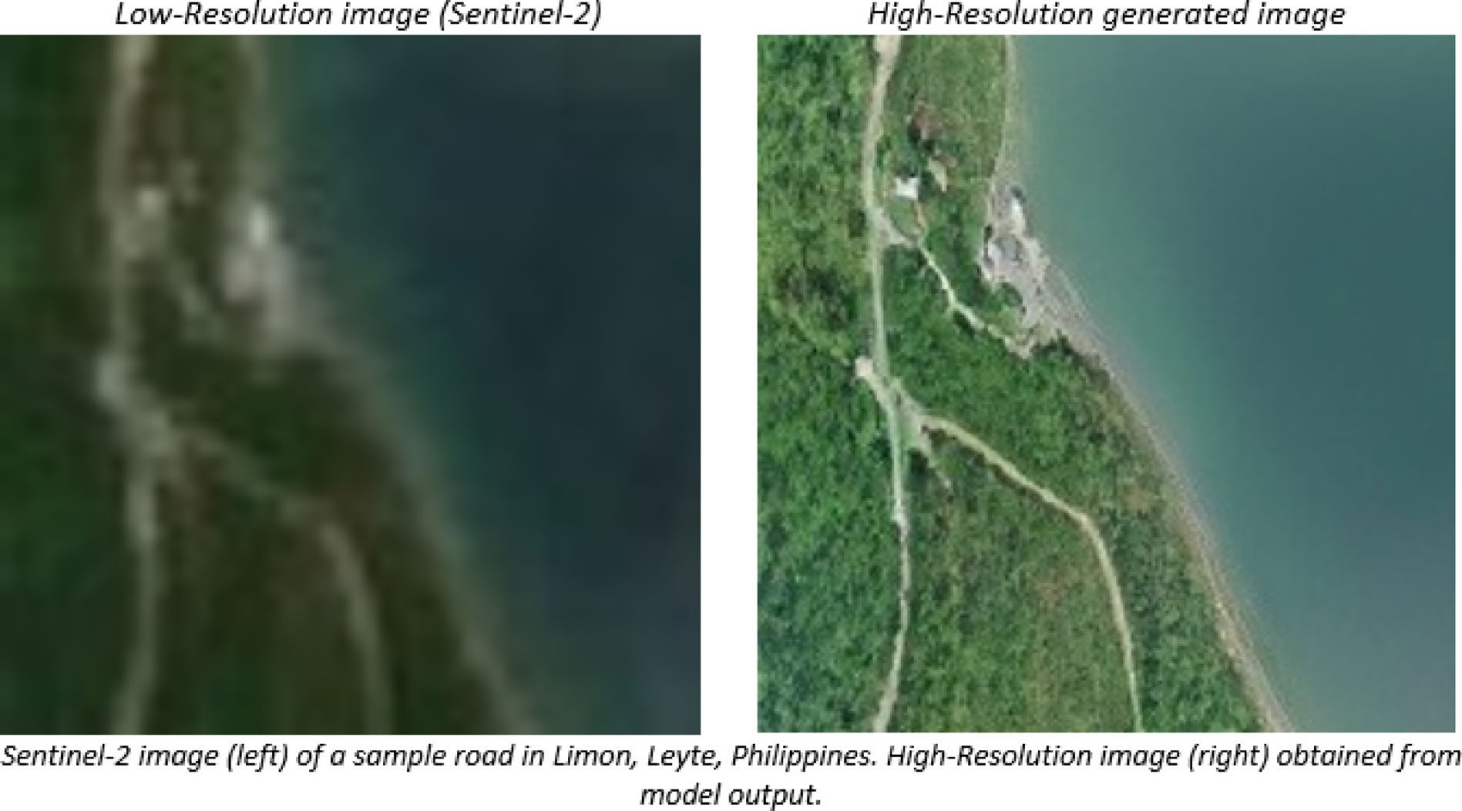
Low-resolution road segment within the Philippines and high-resolution generated image of the same road segment. Sentinel-2 image (left) of a sample road in Limon, Leyte, Philippines. High-Resolution image (right) obtained from model output. Source: Low-Resolution image downloaded from Google Earth Engine https://developers.google.com/earth-engine/datasets/catalog/COPERNICUS_S2_SR.

Following validation, the fine-tuned model was applied to the Philippines dataset. For each of the 124,462 road segments, a cloud-free Sentinel-2 mosaic (generated via a 90-day temporal masking algorithm) was processed to generate a synthetic high-resolution image (Fig. 5). A 2.5 km buffer was maintained around each segment to ensure sufficient contextual information for the subsequent classification algorithms.

### Road quality classification

To classify road segments into the four IRI categories, we implemented CNNs using the PyTorch framework. This approach facilitated distributed training on the full dataset of 124,462 images, significantly increasing statistical power compared to previous iterations that relied on subsampling. To ensure our findings were robust to model selection, we benchmarked two distinct architectures initialized with weights pre-trained on ImageNet: ResNet-34 and EfficientNet-B0. ResNet-34 was selected for its balance of depth and trainability, utilizing skip connections to mitigate the vanishing gradient problem and learning complex hierarchical features of road surfaces. Conversely, EfficientNet-B0 was chosen to evaluate whether parameter-efficient, lightweight models derive greater benefit from super-resolved inputs.

For the multi-modal analysis involving both satellite imagery and environmental data, we constructed a dual-head architecture. The visual head consisted of a ResNet-34 backbone for feature extraction from satellite imagery, while the tabular head employed a sequential linear neural network (SLNN) to process normalized environmental variables. The output vectors from both heads were concatenated and passed through a final fully connected fusion layer, incorporating Batch Normalization and Dropout ($\:p=0.5$) to predict the final IRI class.

We implemented CNNs using PyTorch to classify road segments into the four IRI categories. PyTorch facilitated distributed training on the full dataset of 124,462 images and increased statistical power compared to subsampling methods used in prior studies. We benchmarked two standard architectures to ensure the results were not model-specific. First, we employed ResNet-34, a deep residual network chosen for its balance of depth and trainability. Its skip connections mitigate the vanishing gradient problem and allow the network to learn complex hierarchical features of road surfaces. Second, we used EfficientNet-B0 to test whether parameter-efficient models benefit more from super-resolved inputs. Both models were initialized with weights pre-trained on ImageNet to leverage transfer learning. For the multi-modal analysis, we constructed a dual-head architecture comprising a ResNet-34 backbone for visual features and a Sequential Linear Neural Network for tabular environmental variables.

### Implementation and evaluation

All experiments were conducted on 1x NVIDIA RTX 4080 16GB to accommodate the large-scale dataset. To improve generalization and prevent overfitting, the training pipeline incorporated data augmentation techniques, including random horizontal flips (p = 0.5), random rotations (± 10∘), and color jitter (brightness/contrast ± 0.2). We utilized the Adam optimizer with a weight decay of 0.01. The learning rate was initialized at 1 × 10 − 3 and managed via a Cosine Annealing scheduler, which gradually reduced the rate to 1 × 10 − 6 over the training duration. To address the inherent class imbalance, specifically the predominance of ‘Good’ and ‘Fair’ roads, we applied a Cross-Entropy Loss function weighted by the inverse class frequency. We evaluated model performance using Precision, Recall, and F1-Score for each class to account for the imbalanced nature of road quality data, alongside confusion matrices to identify specific misclassification patterns.

## Results

### Super-resolution image quality assessment

We evaluated the perceptual quality of super-resolved imagery by computing the peak signal-to-noise ratio (PSNR) and structural similarity index measure (SSIM) across eight independent trials, each comprising 100 randomly selected image pairs (Table 1). For each low-resolution Sentinel-2 image, we generated high-resolution outputs using Real-ESRGAN and bicubic interpolation, then compared both against ground-truth NAIP imagery.

Real-ESRGAN achieved mean PSNR values ranging from 15.47 to 17.44 dB across trials, compared to 13.46 to 16.37 dB for bicubic interpolation. SSIM values for Real-ESRGAN ranged from 0.24 to 0.33, compared to 0.17 to 0.33 for bicubic interpolation. These metrics confirm that the generative model produces images with improved perceptual quality relative to traditional interpolation. However, as the following sections demonstrate, this improvement did not translate to enhanced classification performance.

**Table 1:** Image quality metrics for bicubic interpolation and real enhanced super-resolution generative adversarial networks super-resolution.

Bicubic interpolation performance			ESRGAN performance	
	PSNR	SSIM	PSNR	SSIM
1	13.46	0.23	16.46	0.25
2	13.88	0.17	15.85	0.26
3	15.78	0.24	15.47	0.24
4	16.12	0.29	16.53	0.29
5	16.37	0.31	17.44	0.33
6	15.83	0.25	17.29	0.28
7	15.20	0.33	17.27	0.3
8	15.48	0.33	17.31	0.31

PSNR = peak signal-to-noise ratio, SSIM = structural similarity index measure.

Source: Authors’ model performance evaluation.

### Classification performance: super-resolution comparison

Table 2 presents the classification accuracy for road quality assessment using low-resolution Sentinel-2 imagery (10 m per pixel) and super-resolved outputs scaled by a factor of 4. We evaluated two CNN architectures (EfficientNet-B0 and ResNet-34) across binary (Good/Bad) and four-class (Good, Fair, Poor, Bad) classification tasks, training each model for 20 epochs.

**Table 2:** Classification accuracy of low-resolution and high-resolution images using EfficientNet-B0 and ResNet-34 (%).

	EfficientNet-B0 network (%)		ResNet-34 network (%)	
	Binary	4-class	Binary	4-class
Low resolution (Sentinel-2)	79.75	57.59	80.29	53.32
High resolution (Real-ESRGAN)	80.16	55.24	80.16	49.40
High resolution (bi-cubic interpolation)	70.99	44.89	67.89	44.19

Source: Authors’ model predictions.

For binary classification, super-resolution produced negligible accuracy changes. EfficientNet-B0 achieved 79.75% on Sentinel-2 images compared to 80.16% on Real-ESRGAN outputs, a difference of 0.41% points. ResNet-34 showed a slight decrease from 80.29% to 80.16%. For four-class classification, super-resolution reduced accuracy. EfficientNet-B0 declined from 57.59 to 55.24%, while ResNet-34 declined from 53.32 to 49.40%. Bicubic interpolation performed substantially worse across all conditions, with binary accuracy below 71% and four-class accuracy below 45%. These patterns were consistent across both neural network architectures, though EfficientNet-B0 generally outperformed ResNet-34 for four-class classification.

### F1 scores and recall

Tables 3 and 4 present F1 scores and recall values, providing additional insight into model performance beyond overall accuracy.

**Table 3:** F1 scores of low-resolution and high-resolution images using ResNet-34 and EfficientNet-B0 (%).

	ResNet34		EfficientNetB0	
	Binary	4-class	Binary	4-class
Low resolution (Sentinel-2)	78.65	51.11	78.71	54.08
High resolution (Real-ESRGAN)	78.20	49.11	78.15	55.35

Notes & Sources: Authors’ model predictions.

**Table 4:** Recall scores of low-resolution and high-resolution images using ResNet-34 and EfficientNet-B0 (%).

	ResNet34		EfficientNetB0	
	Binary	4-class	Binary	4-class
Low resolution (Sentinel-2)	84.32	51.36	80.00	55.49
High resolution (Real-ESRGAN)	83.18	50.00	78.92	55.14

For binary classification, super-resolution produced negligible accuracy changes. Efficient The F1 scores and recall values are consistent with the accuracy findings. For ResNet-34, binary F1 decreased from 78.65 to 78.20% when using super-resolved imagery, and four-class F1 decreased from 51.11 to 49.11%. Recall showed similar patterns, with binary recall declining from 84.32% to 83.18% and four-class recall declining from 51.36% to 50.00%. For EfficientNet-B0, binary metrics showed modest declines (F1 from 78.71% to 78.15%; recall from 80.00 to 78.92%), while four-class metrics remained relatively stable. These results indicate that super-resolution does not improve the model’s ability to correctly identify road quality classes.

### Confusion matrix analysis: binary classification

**Fig. 6:**
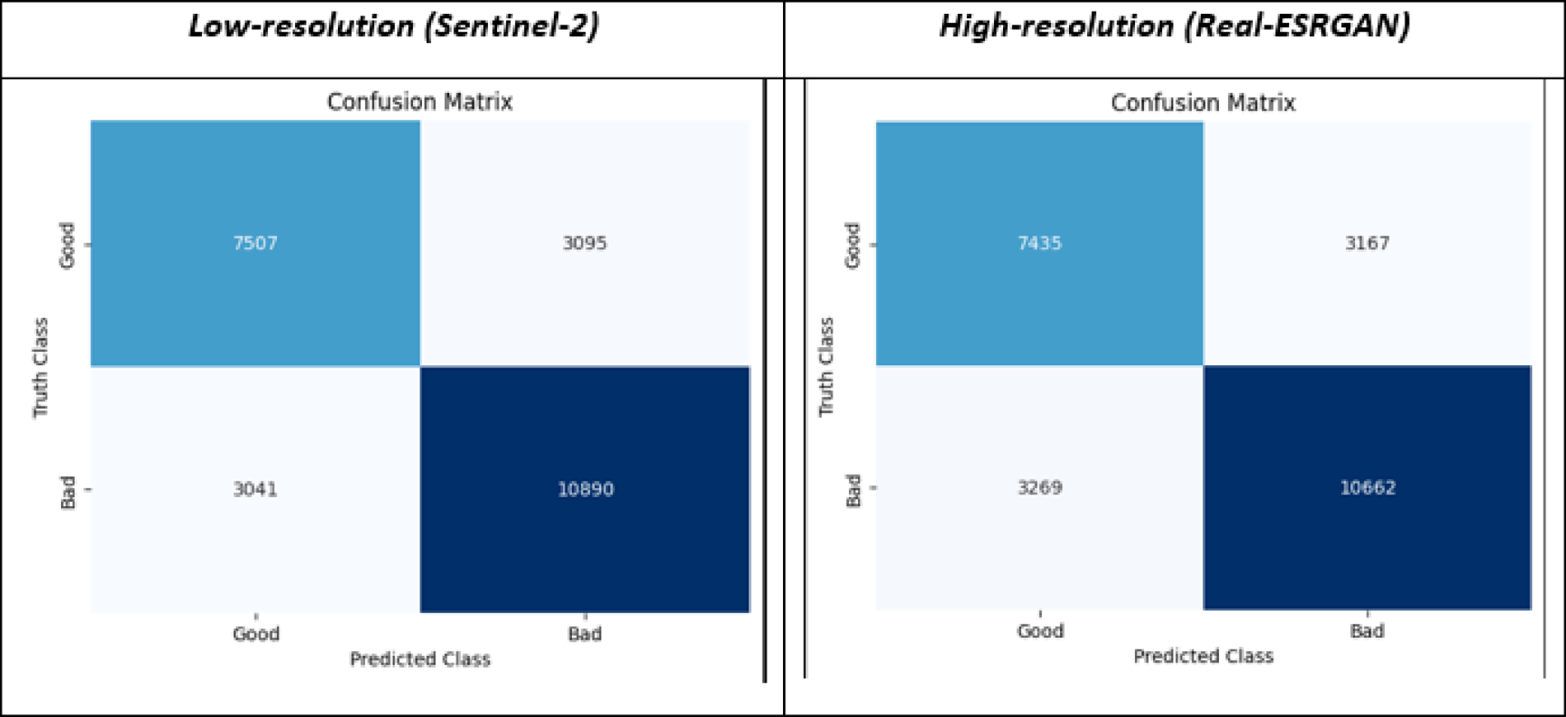
Confusion matrices of low-resolution and high-resolution images using ResNet34 network (percent) binary class.

We evaluated model performance using confusion matrices comparing predicted labels with ground-truth classes. Figure 6 presents results for binary classification using ResNet-34 on both image types.

For low-resolution Sentinel-2 imagery, the model correctly classified 7,507 Good instances and 10,890 Bad instances. Misclassifications occurred in both directions: 3,095 Good samples were incorrectly predicted as Bad, and 3,041 Bad samples were predicted as Good. The total sample size was 24,533, yielding 18,397 correct classifications and 6,136 errors. The model demonstrated stronger performance for the Bad class, with a true positive rate of 78.2% compared to 70.8% for the Good class.

For Real-ESRGAN imagery, the model correctly classified 7,435 Good instances and 10,662 Bad instances. Misclassifications increased: 3,167 Good samples were incorrectly predicted as Bad, and 3,269 Bad samples were predicted as Good. The total number of errors increased from 6,136 to 6,436, representing a 4.9% increase in misclassification when using super-resolved imagery. This indicates that super-resolution did not improve classification performance and marginally degraded it.

**Fig. 7:**
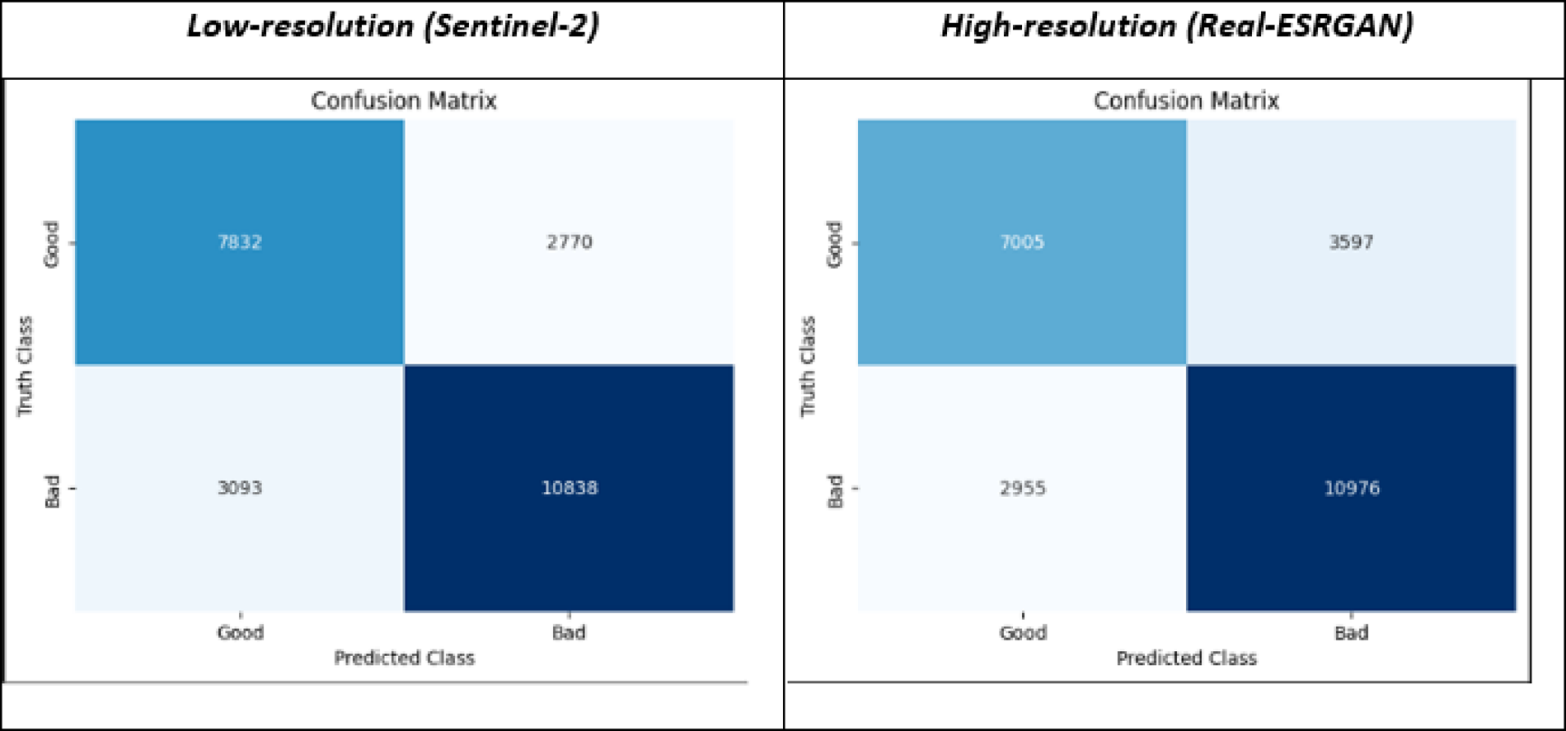
Confusion matrices of low-resolution and high-resolution images using EfficientNet-B0 network (percent) binary class.

Figure 7 presents binary classification results for EfficientNet-B0. For Sentinel-2 imagery, the model correctly classified 7,832 Good instances and 10,838 Bad instances, with 2,770 Good samples misclassified as Bad and 3,093 Bad samples misclassified as Good. For Real-ESRGAN imagery, the model correctly classified 7,005 Good instances and 10,976 Bad instances, with 3,597 Good samples misclassified as Bad and 2,955 Bad samples misclassified as Good. The shift in error distribution indicates that super-resolution increased false negatives for the Good class while slightly reducing false negatives for the Bad class, with no net improvement in overall accuracy.

### Confusion matrix analysis: four-class classification

**Fig. 8:**
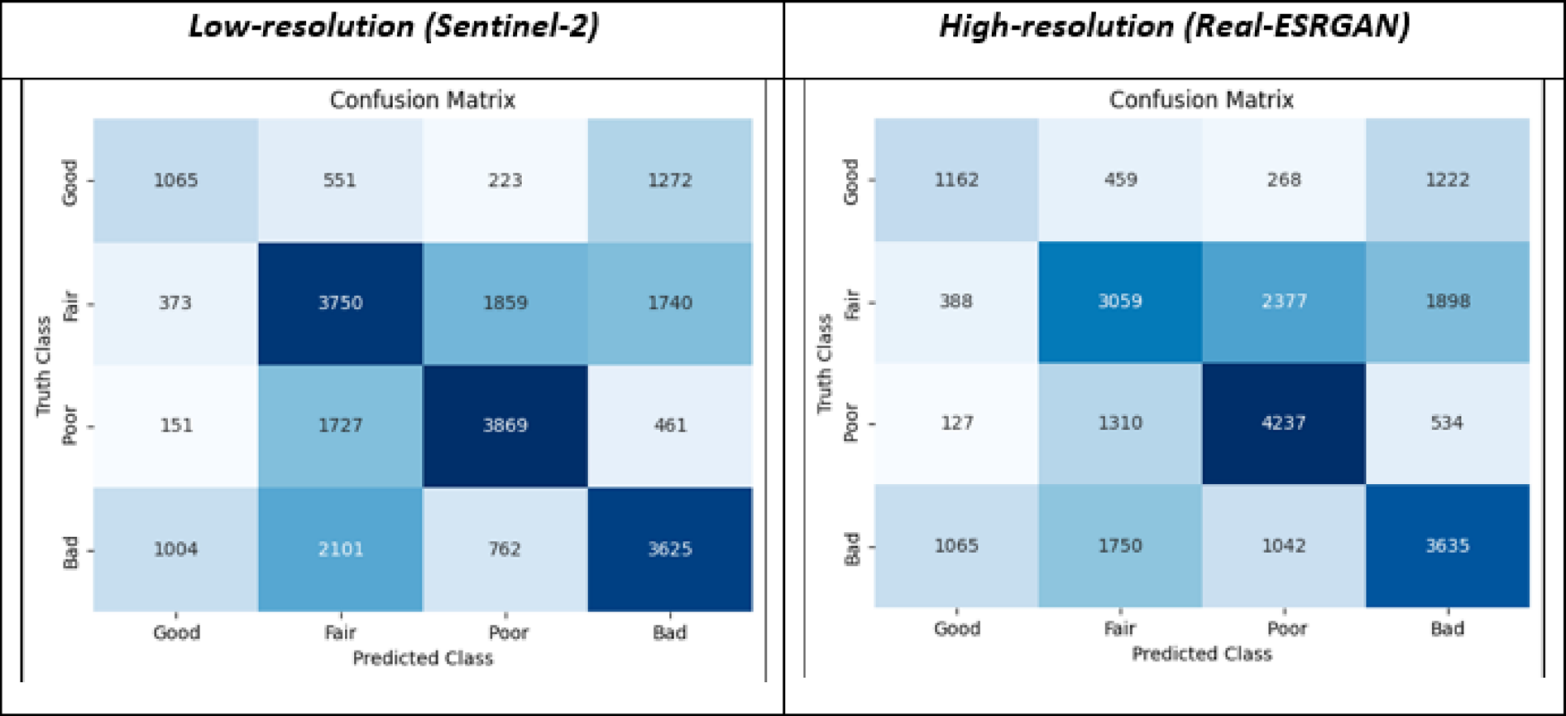
Confusion matrices of low-resolution and high-resolution images using ResNet34 network (percent) four class.

Figure 8 presents four-class classification results for ResNet-34. For Sentinel-2 imagery, the model achieved the following correct classifications: Good (1,065), Fair (3,750), Poor (3,869), and Bad (3,625). Misclassifications revealed systematic patterns of adjacent-class confusion. For the Good class, 1,272 samples were predicted as Bad and 551 as Fair. Fair samples were most frequently misclassified as Poor (1,859) or Bad (1,740). Poor instances showed confusion primarily with Fair (1,727), with smaller numbers assigned to Bad (461). For the Bad class, 2,101 samples were predicted as Fair and 1,004 as Good.

For Real-ESRGAN imagery, correct classifications were: Good (1,162), Fair (3,059), Poor (4,237), and Bad (3,635). The model showed reduced accuracy for the Fair class (3,059 vs. 3,750 correct) while the Poor class showed improved detection (4,237 vs. 3,869 correct). However, overall accuracy declined due to increased inter-class confusion. Fair samples showed greater misclassification as Poor (2,377 vs. 1,859) and Bad (1,898 vs. 1,740). For the Bad class, 1,750 samples were predicted as Fair, 1,042 as Poor, and 1,065 as Good.

The confusion matrices reveal that misclassifications predominantly occur between adjacent IRI classes. For four-class models, approximately 73% of errors involved confusion between neighboring categories (Good-Fair, Fair-Poor, or Poor-Bad). This suggests that CNN captures the ordinal nature of road quality but struggles to distinguish between conditions with similar roughness values. Super-resolution did not reduce this adjacent-class confusion; in the case of ResNet-34, it increased Fair-Poor confusion by 27.9% (from 1,859 to 2,377 misclassifications).

**Fig. 9:**
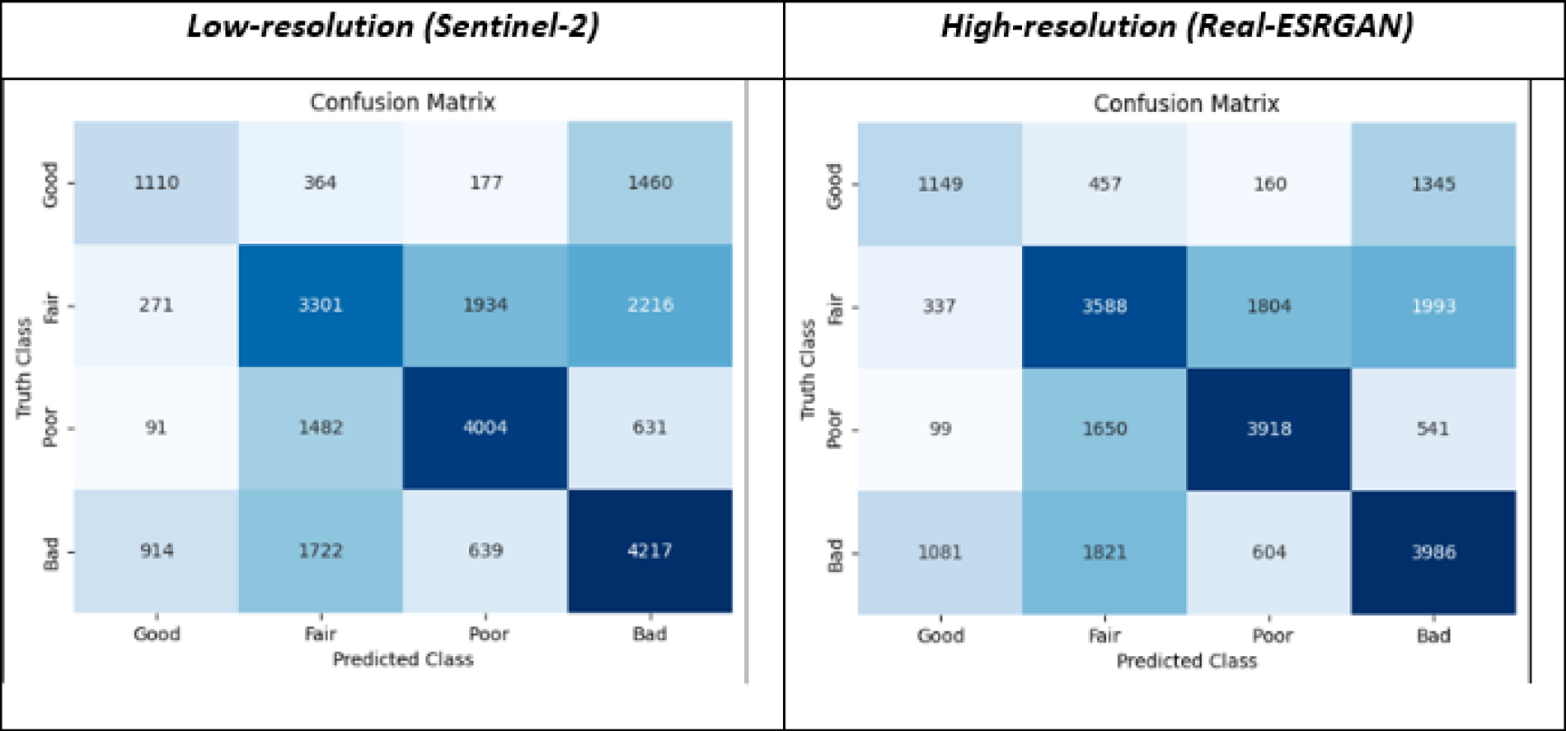
Confusion matrices of low-resolution and high-resolution images using EfficientNet-B0 network (percent) four class.

Figure 9 presents the four-class classification performance of EfficientNet-B0 using Sentinel-2 imagery and Real-ESRGAN. For Sentinel-2, the model correctly classified 1,110 samples as Good, 3,301 as Fair, 4,004 as Poor, and 4,217 as Bad. The matrix reveals substantial inter-class mixing, characterized by strong dispersion across both adjacent and extreme categories. Good samples were most frequently misclassified as Bad (1,460), followed by Fair (364) and Poor (177). Fair instances show extensive confusion with Bad (2,216) and Poor (1,934). Poor samples were primarily misclassified as Fair (1,482), with fewer assignments to Bad (631) and Good (91). Bad samples were most often confused with Fair (1,722), with additional confusion towards Good (914) and Poor (639).

For the Real-ESRGAN enhanced imagery, correct classifications increased to 1,149 for Good, 3,588 for Fair, 3,918 for Poor, and 3,986 for Bad. Although inter-class confusion persists, the misclassification structure shows a more organized pattern dominated by adjacent-class errors rather than extreme cross-class assignments. Good samples were most often misclassified as Bad (1,345) and Fair (457), while Fair samples were mainly confused with Bad (1,993) and Poor (1,804). Poor instances continued to exhibit strong confusion with Fair (1,650), with fewer misassignments to Bad (541) and Good (99). Bad samples were predominantly misclassified as Fair (1,821), followed by Good (1,081) and Poor (604).

### Classification performance by architecture

Table 5 compares classification accuracy across CNN architectures trained on the full dataset of 124,462 road segments using native Sentinel-2 imagery.

**Table 5:** Classification accuracy by convolutional neural network architecture (%).

Architecture	Binary	4-class
ResNet 34	79.6	54.8
ResNet 50	77.3	55.2
EfficientNet B0	81.5	61.5

### Combined model results

EfficientNet-B0 achieved the highest accuracy for both binary (81.5%) and four-class (61.5%) classification. ResNet-34 and ResNet-50 showed comparable performance, with ResNet-34 achieving marginally higher binary accuracy (79.6 vs. 77.3%) while ResNet-50 achieved slightly higher four-class accuracy (55.2 vs. 54.8%). The superior performance of EfficientNet-B0 is consistent with its compound scaling approach, which balances network depth, width, and resolution for improved feature extraction efficiency.

#### Combined image and tabular model

Table 6 presents classification accuracy for the combined model, which integrates satellite imagery with environmental tabular variables including temperature, precipitation, slope gradient, and population density.

**Table 6:** Classification accuracy of combined image-tabular model (%).

	Binary	4-class
Image only	81.5	61.5
Combined (Image + Tabular)	85.0	71.1

The combined model achieved 85.0% binary accuracy and 71.1% four-class accuracy, representing improvements of 3.5 and 9.6% points over the image-only model, respectively. The substantial improvement in four-class accuracy indicates that environmental variables provide discriminative information complementary to visual features. Temperature and precipitation data capture climate-related degradation patterns, slope gradient reflects drainage conditions affecting road wear, and population density correlates with traffic volume and maintenance frequency. These auxiliary variables proved more effective at improving classification than super-resolution enhancement.

For four-class classification, super-resolution reduced accuracy. EfficientNet-B0 accuracy decreased from 57.59 to 55.24% (a reduction of 2.35% points), while ResNet-34 accuracy declined from 53.32 to 49.40% (a reduction of 3.92% points). Bicubic interpolation performed substantially worse than both Sentinel-2 and Real-ESRGAN across all conditions, with binary accuracy below 71% and four-class accuracy below 45%.

For super-resolution, it is notable that the accuracy, especially for the 4-class classification (Good, Fair, Bad, Poor), is generally low. Following this, it is observed that the use of Real-ESRGAN images does not significantly enhance the accuracy.

Table 7 gives the results for training the EfficientNet-B0 for 20 epochs, for the four-class and two-class models. A comparison is given for Sentinel-2 images, which have a resolution of 10 m per pixel, and the super-resolved images generated by the Real-ESRGAN and bi-cubic interpolation, which are scaled by a factor of 4. For comparison, Table 7 also shows the results of the classification models ran with ResNet-34. The same classification patterns are observed across both types of neural networks, although in general, classification accuracies are lower for ResNet than EfficientNet models for the four-class.

**Table 7:** Classification accuracy of low-resolution (LR) and high-resolution (HR) images using EfficientNet-B0 network (%) and ResNet-34 network (%).

	EfficientNet-B0 network (%)		ResNet-34 network (%)	
	Binary	4-class	Binary	4-class
LR (Sentinel-2)	79.75	57.59	80.29	53.32
HR (Real-ESRGAN)	80.16	55.24	80.16	49.40
HR (bi-cubic interpolation)	70.99	44.89	67.89	44.19

Source: Authors’ model predictions.

### Summary

The results yield three principal findings. First, super-resolution via Real-ESRGAN did not improve road quality classification accuracy despite generating images with superior perceptual quality. Binary classification accuracy remained unchanged (within 0.5% points), while four-class accuracy decreased by 2.4 to 3.9% points depending on architecture. Second, among the architectures evaluated, EfficientNet-B0 achieved the highest performance on native Sentinel-2 imagery, with 81.5% binary and 61.5% four-class accuracy. Third, the combined image-tabular model achieved 85.0% binary and 71.1% four-class accuracy, demonstrating that environmental context variables provide greater discriminative value than generative image enhancement. These findings indicate that preserving authentic spectral information from medium-resolution satellite imagery, augmented with environmental data, provides a more effective approach for road quality assessment than super-resolution reconstruction.

## Discussion

Satellite imagery combined with deep learning presents a scalable approach for road quality monitoring in resource-constrained settings. This study investigated whether super-resolution techniques could enhance the utility of freely available medium-resolution imagery for road roughness classification. Our findings indicate that generative image enhancement does not improve classification accuracy and may introduce artifacts that degrade model performance.

The core finding of this study is that Real-ESRGAN super-resolution yielded no improvement in road quality classification despite producing images with superior perceptual quality. Binary classification accuracy remained within 0.5% points between Sentinel-2 and super-resolved inputs, while four-class accuracy decreased by 2.4 to 3.9% points. This result contradicts the intuitive assumption that higher resolution imagery should improve classification performance.

Several factors may explain why super-resolution failed to enhance discriminative accuracy. First, GANs address an ill-posed inverse problem by inferring plausible high-frequency details from low-resolution inputs. The generated textures are optimized for perceptual similarity to natural images rather than for preserving diagnostic features relevant to road surface condition. The model predicts details that appear realistic but carry no information about actual pavement roughness. Second, the 10-meter native resolution of Sentinel-2 cannot capture the millimeter-scale surface irregularities that determine International Roughness Index values. Super-resolution cannot recover physical information that was never recorded by the sensor. Third, our classification architecture incorporates a 2.5-kilometer buffer around each road segment, meaning that contextual features such as land use patterns, vegetation, and urban density contribute substantially to predictions. These contextual features are already well-represented at medium resolution, and adding synthetic texture to road surfaces provides minimal additional signal.

The domain gap between training and application data likely compounded these limitations. The Real-ESRGAN model was fine-tuned on paired imagery from United States road networks using NAIP ground truth, then applied to Philippine roads. Road construction materials, lane widths, shoulder characteristics, and surrounding land cover differ substantially between these regions. The GAN may generate textures appropriate for US road surfaces but inappropriate for Philippine conditions, introducing systematic errors. Additionally, tropical climatic conditions in the Philippines produce different degradation patterns than temperate US climates, including moisture-related damage and vegetation encroachment that the model was not trained to represent.

The confusion matrix analysis revealed that misclassifications predominantly occurred between adjacent IRI classes, with approximately 73% of errors involving neighboring categories. This pattern suggests that the CNN captures the ordinal structure of road quality but cannot reliably distinguish between conditions with similar roughness values. Super-resolution did not reduce this adjacent-class confusion. For ResNet-34, Fair-Poor misclassifications increased by 27.9% when using super-resolved imagery, indicating that generative enhancement may amplify boundary ambiguity rather than resolve it.

In contrast to super-resolution, the combined image-tabular model achieved substantial accuracy improvements. By integrating environmental variables including temperature, precipitation, slope gradient, and population density, four-class accuracy increased from 61.5% to 71.1%. This 9.6% point improvement exceeds any variation observed from image resolution changes. Temperature and precipitation data capture climate-driven degradation mechanisms. Slope gradient influences drainage and erosion patterns affecting road wear. Population density correlates with traffic volume and maintenance investment. These auxiliary variables provide physically meaningful information about road condition drivers that cannot be inferred from visual appearance alone.

The practical implication of these findings is that for infrastructure monitoring applications, resources should be directed toward acquiring complementary geospatial data rather than computationally expensive image enhancement – particularly if relevant training super-resolution data is not readily available. The combined model achieving 85% binary accuracy and 71% four-class accuracy represents a viable tool for preliminary identification of road segments requiring intervention. This performance level enables prioritization of field inspections and maintenance resources, particularly valuable in data-sparse regions where traditional road surveys are infrequent.

Several limitations warrant consideration. The classification models were trained and evaluated on Philippine road networks, and generalization to other geographic contexts requires validation. The IRI ground truth data reflects conditions as of 2019, and temporal alignment between survey data and satellite imagery introduces potential measurement error. The four-class accuracy of 71% indicates that approximately 29% of segments would be misclassified, necessitating human verification before maintenance decisions. Future work should investigate domain adaptation techniques for super-resolution models, assess whether alternative super-resolution architectures such as SwinIR and HAT yield different downstream classification results, evaluate transformer-based architectures that may better capture long-range spatial dependencies, and assess temporal change detection for monitoring road degradation over time.

These findings contribute to the growing literature on remote sensing for infrastructure assessment by providing empirical evidence against a commonly hypothesized improvement pathway. For practitioners in developing regions seeking cost-effective road monitoring solutions, our results suggest that native medium-resolution imagery combined with environmental covariates offers a more reliable approach than generative image enhancement.

## Supplementary Information

Supplementary Information.

## Data Availability

The IRI data used and analyzed in the study are owned and provided by the Philippine Department of Public Works and Highways upon official request. Meanwhile, the satellite imagery and other modeled data were obtained from Google Earth Engine (https://earthengine.google.com).
