# Supplementary material for: Evaluating the effectiveness of satellite image super-resolution for road quality monitoring
Source: Sci Rep. 2026 Apr 16;16:17759. doi: 10.1038/s41598-026-47749-3 (PMC13246733; doi:10.1038/s41598-026-47749-3)

# Supplementary Information S1

1. **Model Architectures and Specifications**

The satellite imagery was upscaled and super-resolved using the Real-ESRGAN architecture, a state-of-the-art GAN built upon the Enhanced Super-Resolution GAN (ESRGAN) framework. The generator consists of multiple Residual-in-Residual Dense Blocks (RRDB), which are critical for preserving fine texture details and geometric edges during the upsampling process. The implementation utilizes the Basic Super Restoration (BasicSR) pipeline, an open-source PyTorch toolbox that provides high-level abstractions for the generator and discriminator classes, as well as the adversarial training loop. This pipeline ensures a robust framework for handling super-resolution, denoising, and deblurring tasks.

1. **Convolutional Neural Network (CNN) Backbones**

For the image classification modality, we employed two distinct backbone architectures, both pre-trained on ImageNet to leverage transfer learning. The first is ResNet-34, a deep convolutional network comprised of Basic Residual Blocks. The final fully connected layer was modified to output four neurons, corresponding to the four road quality classes (Good, Fair, Poor, Bad). The second architecture is EfficientNet-B0, which is comprised of Mobile Inverted Bottleneck Blocks (MBConv) and was similarly modified at the final fully connected layer to output four neurons.

Standard image preprocessing for the vision-based data involved resizing inputs to consistent dimensions of 224*224 pixels, normalization using standard ImageNet means (μ=[0.485, 0.456, 0.406]) and standard deviations (σ=[0.229, 0.224, 0.225]), and conversion to PyTorch tensors.

1. **Dataset Preparation**

The image dataset underwent a rigorous preprocessing pipeline. All input images were resized to a resolution of 224*224 pixels and normalized. To ensure robust evaluation, the dataset was divided into training (80%) and testing (20%) subsets using a stratified split strategy (sklearn.model_selection.train_test_split), ensuring that the class distribution remained consistent across both sets. Categorical labels were processed using One-Hot Encoding via PyTorch functional utilities.

1. **Data Loading and Hyperparameters**

We utilized the standard PyTorch Data Loader (torch.utils.data.DataLoader) to combine the image dataset with a sampler, producing an iterable object optimized for efficient memory management. To maximize input/output throughput, 64 worker processes were allocated to the Data Loader. Due to the high memory requirements of processing super-resolved imagery, a batch size of 4 was strictly enforced.

The training process was governed by the Cross-Entropy Loss function (torch.nn.CrossEntropyLoss), which was weighted by the inverse class frequency to mitigate the effects of dataset imbalance. Optimization was performed using the **Adam** optimizer (torch.optim.Adam) with a learning rate of $1\times{10}^{-3}$ and a weight decay of $1\times{10}^{-2}$. The optimizer’s computation graph was initialized using the CUDA backend to leverage GPU acceleration.

1. **Computational Environment**

The experimental architecture required significant GPU memory to process the combined image and tabular data streams. All training and inference tasks were conducted on a high-performance computing cluster running Ubuntu Linux, which is required to support OpenAI Triton for optimized deep learning execution. The hardware configuration included a single **NVIDIA RTX 4080 GPU** (16GB VRAM), utilizing the ADA Lovelace architecture to support bfloat16 mixed-precision operations for enhanced efficiency. The software environment was standardized on Python 3.11.0 and PyTorch 2.3.0. Under these specifications, the total training duration was approximately four days.

# Supplementary Information S2

1. **Error Maps**

In our analysis, we train our model on high-resolution images from NAIP, paired with low-resolution images from Sentinel satellites. Error maps are computed as the per-pixel absolute difference between super-resolved output and the ground truth, averaged across RGB channels and normalized to a 0 - 1 scale, where darker regions indicate low reconstruction error and brighter regions indicate greater discrepancy. The exercise is performed on ten validation pairs, as illustrated below. Across pairs, reconstruction error is predominantly low, with an average absolute difference of 0.11, confirming that Real-ESRGAN recovers most of the spatial detail accurately. Higher error is concentrated at structural boundaries road edges, building outlines, and fine linear features. This is consistent with the known behavior of GAN-based SR models, where edges and high-frequency details present multiple plausible reconstructions from a single low-resolution input.


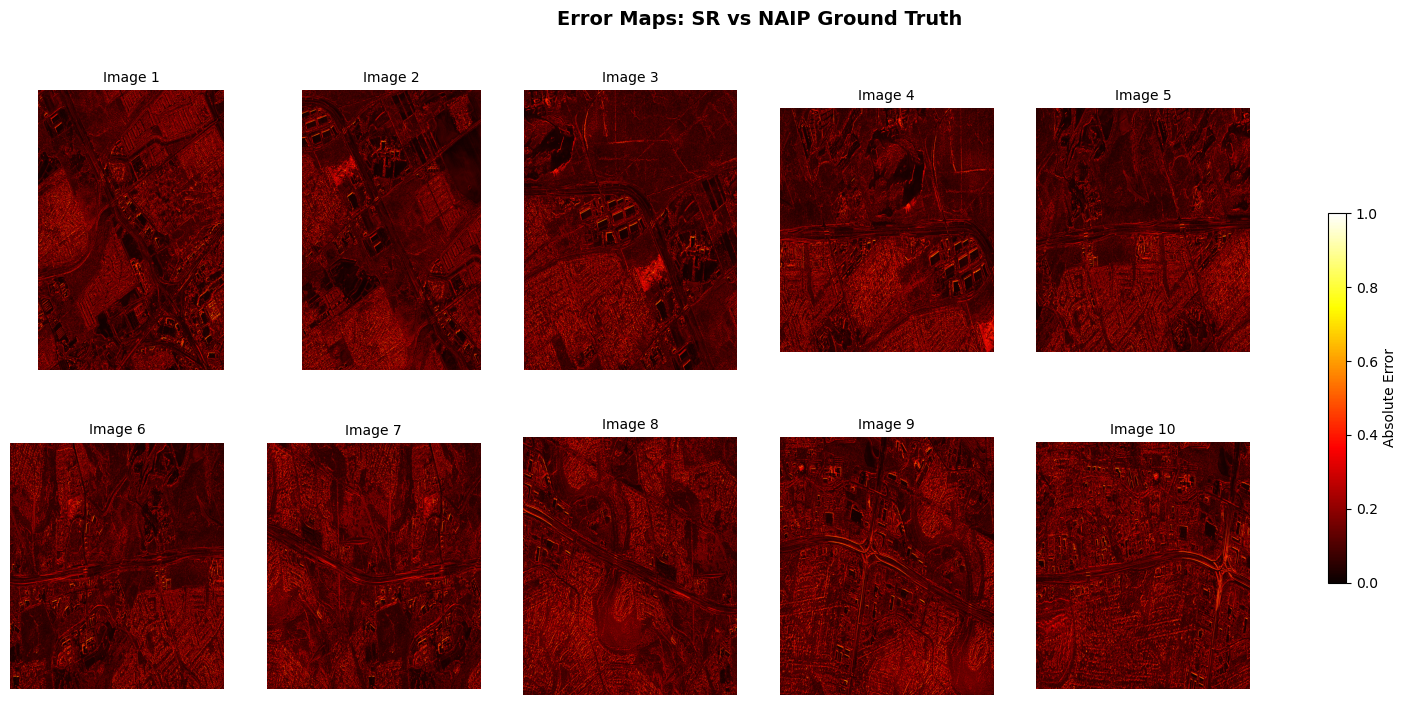

Supplement: Supplementary file 1 — Supplementary Information. [file 41598_2026_47749_MOESM1_ESM.docx]
